# Fatal cardiac air embolism after CT-guided percutaneous needle lung biopsy: medical complication or medical malpractice?

**DOI:** 10.1007/s12024-023-00639-w

**Published:** 2023-05-09

**Authors:** Nicola Pigaiani, Giulio Barbiero, Elisabetta Balestro, Francesco Ausania, Brandi McCleskey, Erica Begni, Federica Bortolotti, Matteo Brunelli, Domenico De Leo

**Affiliations:** 1 Unit of Forensic Medicine, Department of Diagnostics and Public Health, University of Verona, Piazzale L.A. Scuro 10, Verona, 37134 Italy; 2 Division of Forensics, Department of Pathology, University of Alabama at Birmingham, 1515 6th Avenue South, Room 220, Birmingham, AL 35233 USA; 3 Unit of Radiology, Department of Medicine, University of Padova, University Hospital, 35128 Padua, Italy; 4 Unit of Respiratory Diseases, Department of Cardiac, Thoracic, Vascular Sciences and Public Health, University of Padova, University Hospital, 35128 Padua, Italy; 5 Unit of Pathology, Department of Diagnostics and Public Health, University of Verona, Piazzale L.A. Scuro 10, Verona, 37134 Italy

**Keywords:** Fatal cardiac air embolism, CT-guided percutaneous needle biopsy, Lung biopsy, Complications, Forensic pathology

## Abstract

Computed tomography (CT)-guided percutaneous needle biopsy of the lung is a well-recognized and relatively safe diagnostic procedure for suspicious lung masses. Systemic air embolism (SAE) is a rare complication of transthoracic percutaneous lung biopsies. Herein, we present a case of an 81-year-old man who underwent CT-guided percutaneous needle biopsy of a suspicious nodule in the lower lobe of the right lung. Shortly after the procedure, the patient coughed up blood which prompted repeat CT imaging. He was found to have a massive cardiac air embolism. The patient became unresponsive and, despite resuscitation efforts, was pronounced dead. The pathophysiology, risk factors, clinical features, radiological evidence, and autopsy findings associated with SAE are discussed, which may, in light of the current literature, assist with the dilemma between assessing procedural complications and medical liability. Given the instances of SAE in the setting of long operative procedures despite careful technical execution, providing accurate and in-depth information, including procedure-related risks, even the rarest but potentially fatal ones, is recommended for informed consent to reduce medicolegal litigation issues.

## Introduction

Computed tomography (CT)-guided percutaneous needle biopsy of the lung is a diagnostic procedure for suspicious lung lesions. This diagnostic approach is performed in all the cases in which the assessment of the lesion utilizing lung endoscopy is not possible. CT-guided percutaneous needle biopsy has a sensitivity of 93–98% and a specificity of 98–100% for the diagnosis of malignancy and provides critical information for further clinical and surgical management, particularly with current therapies that can be tailored to pathological features and the patient [1]. The procedure is widely accepted as a safe diagnostic tool since most complications, such as pneumothorax, pulmonary bleeding, and hemoptysis, are uncommon and can usually be treated conservatively with favorable outcomes [2, 3]. On the other hand, systemic air embolism (SAE), which occurs when air is introduced into the coronary and/or cerebral arterial vasculature, is a rare but potentially fatal complication. The reported incidence of SAE is 0.08% [4], although the incidence may be higher (reaching almost 5%) due to missed diagnoses in asymptomatic patients [5].

Herein, we report a case of a fatal cardiac air embolism after a percutaneous CT-guided transthoracic needle biopsy performed for a suspicious lung lesion. The clinical, radiological, and pathological findings are presented and discussed. A review of the current literature is also provided, which may aid in solving the dilemma between procedural complications and medical liability.

## Case report

An 81-year-old man was found to have a 1.6 × 1.1 cm nodule in the posterior-basal segment of the lower lobe of the right lung with associated mediastinal lymphadenopathy of CT imaging of the chest. His medical history was pertinent for a diagnosis of squamous cell carcinoma of the lung as well as an intraductal papillary mucinous neoplasm of the pancreas, both of which had been previously treated surgically. Two months after the lesion was identified on CT, the patient underwent 18 F-fluorodeoxyglucose positron emission tomography/computed tomography (18 F-FDG PET/CT), which showed hypercaptation (increased uptake) within the nodule and the left bronchial lymph nodes. After discussion among the multidisciplinary team, the patient underwent bronchoscopy with associated endobronchial ultrasound-guided transbronchial needle aspiration (EBUS-TBNA) of the enlarged lymph nodes. However, the cytological analysis was not diagnostic, with no cellular atypia identified within the submitted material.

**Fig. 1:**
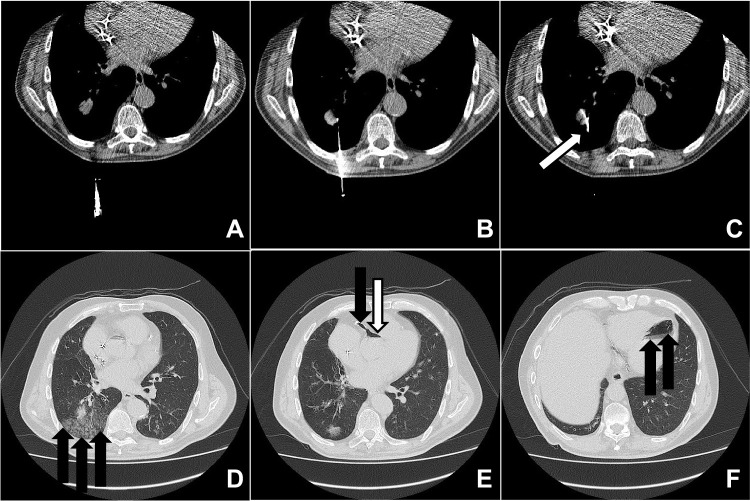
CT scans in sequence obtained during the procedure (**A**, **B**, and **C**) and sequential slices of scan performed after the procedure (**D**, **E**, and **F**). **A** First image in the sequence showing the lesion (light gray mass) within the right lung lobe. **B**  Image in the sequence showing needle being inserted through the skin of the back. **C** Image showing the 17-gauge BioPince-type needle within the lesion (indicated by the white arrow). **D** Image showing hemorrhage along the posterior aspect of the right lung (black arrows). **E** Image showing air within the aorta (white arrow) and right coronary artery (black arrow). **F** Image showing air within the left ventricle (black arrows)

Four months later, a thoracic CT showed that the lung lesion had grown larger (now 2.7 × 1.7 cm). The clinical team decided that a CT-guided percutaneous needle lung biopsy was indicated to diagnose the lesion further. The patient consented to the procedure after a thorough discussion of the associated risks and benefits, along with alternative diagnostic methods, such as surgical lung biopsy and continued imaging surveillance. The procedure was performed in the typical fashion with the patient in the prone decubitus position. Once the lesion was localized under CT, a 17-gauge BioPince-type needle (typically used as a core biopsy instrument) was placed into the lesion on the first pass during a single inspiratory breath-hold (Fig. 1A–C). This needle remained in this position until the end of the procedure, as confirmed with multiple consecutive scans. A coaxial 18-gauge needle was then inserted through the 17-gauge needle to obtain multiple “cutting-type” biopsy samples of the lesion. The insertion of the needle and the biopsies were performed only during inspiratory breath-holding. At the conclusion of the procedure, the 17-gauge needle was removed.

At the end of the procedure, the patient coughed, and bright red blood was expectorated. Of note, the patient did not cough or breathe improperly during the procedure while the needles were inserted. Repeat CT imaging of the chest showed slight hemorrhage around the lesion and a massive air embolism within the left ventricular chamber with extension into the aortic root and right coronary artery (Fig. 1D–F). Unfortunately, the patient suddenly became unresponsive shortly after the CT imaging was obtained and experienced cardiac arrest. After 40 min of adequate resuscitation efforts, the patient was pronounced dead in the radiology suite.

### Autopsy findings

**Fig. 2:**
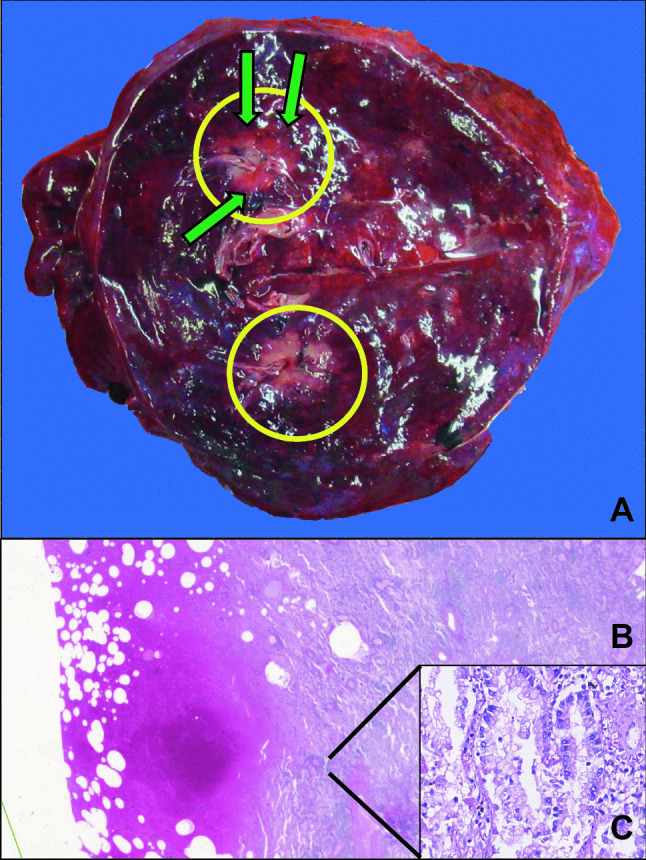
Right lung, lower lobe. **A** Solid white-tan ill-defined mass (yellow circles) surrounded by a hemorrhagic area (green arrows). **B** Hemorrhage and pneumatosis foci with adjacent adenocarcinoma (hematoxylin and eosin stain, 5× magnification). **C** Adenocarcinoma of the lung (hematoxylin and eosin stain, 40× magnification)

Given the circumstances surrounding the death and the necessity to exclude the possibility of medical malpractice, a medicolegal autopsy was performed the following day. Evidence of previous surgical procedures was noted during the examination, including surgical scars and the absence of the upper lobe of the right lung as well as the spleen and distal pancreas. Examination of the abdominal organs did not yield evidence of further natural disease processes. There were also no noteworthy intracranial findings. Examination of the right lung confirmed evidence of the needle-guided procedure along the pleural surface of the posterior aspect of the lower lobe. Further sectioning of the lung lobe revealed a white-tan ill-defined mass measuring approximately 3 cm within an area of hemorrhage (Fig. 2A). Sections were obtained for microscopic examination, which showed areas of hemorrhage and pneumatosis foci within the lung parenchyma (consistent with the insertion of a needle) adjacent to the tumoral tissue. The tumor was consistent with adenocarcinoma of the lung (Fig. 2B, C).

A specific procedure was utilized to confirm the presence of an air embolism within the heart and coronary arteries as indicated on the post-procedure CT scan. The procedure is well documented by Richter [6] and Zolotarov and Fraser [7] and includes filling the pericardial sac with water and inserting a large bore needle with attached water-filled syringe into each chamber of the heart and the great vessels while watching for bubbles to enter the syringe. In the present case, bubbles appeared in the syringe barrel when the needle tip was inserted into the left ventricle and the ascending aorta. Further examination of the heart did not reveal any significant structural pathological findings. Based on the available clinical data and autopsy findings, the death was attributed to a massive left cardiac and coronary air embolism (found within the left ventricle and extending into the right coronary artery) following the CT-guided percutaneous needle biopsy of the lung.

## Discussion

In the case reported herein, the medical record review showed that CT-guided percutaneous needle biopsy was an indicated diagnostic procedure considering the medical history of the patient, the radiological findings, and the impossibility of formulating a diagnosis of the lung lesion through EBUS-TBNA. Unfortunately, the patient experienced a systemic air embolism (SAE) that directly lead to his death shortly after the procedure. Mortality due to systemic air embolism (SAE) following CT-guided percutaneous needle biopsy of the lung is reported to be approximately 0.0002% in the largest series [8]. To the best of our knowledge and after a thorough review, only seven SAE fatal cases of air embolism have been thoroughly described in the literature [9–15], as summarized in Table 1. While the vast majority of SAEs are undiagnosed in asymptomatic patients, in cases of life-threatening SAE, diagnosis is generally clinically suspected based on the abrupt decline of the neurologic and/or cardiovascular condition of the patient, like that seen in the patient presented. The prognosis of SAE depends primarily on the quantity of air entering the vascular system. Brain and chest CT scans can provide a radiologic confirmation by detecting bubbles (typically seen as a void in the contrast material within the vessel) in the cerebral vascular system, ascending aorta, the left side of the heart, and pulmonary veins [16]. Early treatment of a SAE consists of prompt administration of 100% oxygen and placing the patient in the left lateral decubitus position with lowering of the head in an effort to increase the intracavitary pressure of the left atrium and avoid cerebral embolization of air [17].

**Table 1:** Fatal cases of air embolism complicating CT-guided percutaneous needle biopsy of the lung reported in the literature

**Authors**	**Number of reported cases**	**Gender**	**Age**	**Known patient risk factors**	**Radiological evidence for diagnostic approach**	**Patient position during the procedure**	**Method and needles used**	**Radiologic investigation post-procedure**	**Postmortem examination**
Kodama et al. (1999) [9]	1	M	60	No	Pulmonary nodule with pleural indentation in the right lower lobe	Left lateral	Coaxial 18G introducer 21G aspiration needle	Yes	No
Arnold and Zwiebel (2002) [10]*	1	M	60	Chronic obstructive pulmonary disease	Lung lesion in left lower lobe (size: 2.5 cm)	Right lateral	Coaxial 19G introducer 21G aspiration needle	Yes	Yes
Ghafoori and Varedi (2008) [11]	1	F	50	No	Multiple nodular lesions of lungs	Prone	18G tru-cut needle	Yes	No
Bou-Assaly et al. (2010) [12]	1	M	76	Chronic obstructive pulmonary disease	Pulmonary round mass (size 3.5 cm) in the left upper lobe	Supine	Coaxial 17G introducer 18G core biopsy needle	Yes	No
Sun et al. (2015) [13]	1	M	53	No	Multiple nodular and flake-shaped lesions in both lungs	Prone	Coaxial 17G introducer 18G cutting needle	Yes	No
Ornelas et al. (2018) [14]	1	M	70	No	Pulmonary nodule in the right lung	Left lateral	N.D.	Yes	No
Ialongo et al. (2017) [15]	1	M	57	No	Multiple bilateral pulmonary nodules in adenocarcinoma of the prostate	Prone	21G aspiration needle	Yes	No
Current case for comparison	1	M	80	No	Pulmonary nodule in a patient with a history of lung, and pancreas cancer	Prone	Coaxial 17G introducer 18G cutting needle	Yes	Yes

*N.D.* not declared

*similar presentation to the current case but without medicolegal context

From a pathophysiological point of view, arterial air embolism stems from air entering the pulmonary veins during percutaneous needle biopsy of the lung. According to the literature, several different mechanisms may be responsible for air entry into the pulmonary venous system, firstly, through a hole in the pulmonary vein caused by the needle (catheter) after removal of the inner stylet, resulting in the rising of the pressure gradient between atmospheric pressure and the pulmonary venous pressure (likely occurring during inspiration). In this case, air may enter directly through the catheter. Secondly, air may be directly injected during the procedure into the pulmonary arterial circulation and then enter the pulmonary veins by crossing the pulmonary capillaries. Lastly, the needle may simultaneously penetrate the pulmonary vein and an adjacent air-containing space (i.e., alveolar space, bronchus, air cyst, cavity), creating a communicating fistula. In the latter case, Valsalva maneuvers can increase the pressure in the air space, resulting in vascular air embolism [5, 12]. It is worth mentioning that an air volume of 0.5–1.0 mL is enough to cause cardiac arrest through coronary artery air embolism, and 2.0 mL is enough to cause a fatal stroke through cerebral air embolism [18].

The prevention of SAE includes operator and patent-related measures. The operator must ensure the occlusion of the introducer needle by the inner stylet or own finger, while the patient should be instructed to avoid breathing deeply and coughing during the biopsy [19]. Some authors suggest an increased probability of SAE when using a coaxial approach and larger needles. In fact, the coaxial method increases the risk of communication of the lung parenchyma with the atmosphere after extraction of the inner stylet, whereas larger needles show an increased risk of pulmonary vein puncture along the needle path [20]. However, fatal SAE may also occur using techniques other than the coaxial method [9, 10, 12, 13] and with smaller needles [15]. The patient’s position during the procedure has also been discussed as a potential risk factor for the development of fatal SAE [4]. However, analysis of the literature about SAE cases has shown that a fatal event can occur regardless of the patient’s position during the procedure. Additional potential risk factors mentioned in the literature are post-inflammatory changes in the portion of the lung traversed by the needle, including increased vascularity, vasculitis, or friable lung tissue. All of these could affect the physiologic hemostatic mechanisms resulting in protracted exposure of the blood flow to the airway [5].

To the best of our knowledge, this is the first case of fatal SAE to be discussed within a medicolegal context and with available radiological and autopsy evidence to assist with evaluating operator behavior. In particular, the macroscopic and microscopic analyses of the lung revealed the presence of a distinct and straight pathway through the parenchyma from the skin to the lung mass. This, along with the CT scans obtained during the procedure, supports the adherence of the operator to the international procedural guidelines [17], which recommend choosing the shortest needle path to the lesion to reduce the thickness of the involved parenchyma. In this case, it was obtained by keeping the patient in the prone position and by using a coaxial method that allowed for multiple needle biopsy sampling with a single intraparenchymal path created by the core biopsy needle.

The above considerations ruled out medical errors regarding indication and/or operative technique and further identified the occurrence of cardiac air emboli as an intervention complication. In our opinion, given the clinical and autopsy findings, a significant entry of air through a fistula between the air-containing space (alveolar space) and an adjacent pulmonary venous vessel created during the needle penetration likely occurred. It is likely that the Valsalva maneuver related to the cough of the patient at the end of the procedure facilitated air penetration into the vascular system. Air reached the left heart and coronary arteries through the pulmonary vein causing heart and coronary embolism with resulting myocardial ischemia, decreased myocardial function, and death.

Despite the unfavorable outcome, the multidisciplinary review of the procedure indicated that in the absence of any known risk factors, alternatives such as conservative surveillance through imaging and invasive surgical options both shared significant risks when compared to CT-guided percutaneous needle biopsy. Furthermore, the consent procedure was adequate, and ultimately, the patient was able to make an informed decision regarding his care plan, correctly carried out, giving to the patient all the information needed for his choice.

The medicolegal point of view proposed herein for analyzing fatal SAE cases is important because air embolism following CT-guided percutaneous needle lung biopsy is a complication that is difficult to prevent and could serve as a possible source of litigation. Although several recommendations and precautions have been suggested to reduce the risk of SAE following CT-guided percutaneous needle biopsy of the lung, this complication can occur particularly in cases with long operative exposure and despite careful technical execution. It is often worth acknowledging that in the context of personalized treatment, this diagnostic procedure represents a major trend in the future [21]. On this basis, a thorough disclosure of the procedure given preferably by the operator during the consent process, including all procedure-associated risks, even the rarest but potentially fatal ones, is recommended. In fact, to ensure adequate informed consent, providing accurate and in-depth information, including alternative invasive and conservative approaches, is essential to reduce medicolegal litigation issues.

## Key points

1. Computed tomography (CT)-guided percutaneous needle biopsy of the lung is a safe diagnostic procedure for suspicious lung lesions.
2. Systemic air embolism (SAE) is a rare and potentially fatal procedure-related complication.
3. An 81-year-old man died after SAE development during a CT-guided needle biopsy of the lung.
4. The thorough medicolegal investigation identified SAE as a procedure-related complication excluding medical malpractice.
5. Thorough disclosure of the procedure given, preferably by the operator during the consent process, is recommended in order to avoid medicolegal litigation issues.

## Data Availability

The data analyzed during the current study are available from the corresponding author upon reasonable request.
